# Evaluation of the flying focal spot technology in a wide-angle digital breast tomosynthesis system

**DOI:** 10.1117/1.JMI.12.S1.S13009

**Published:** 2024-12-04

**Authors:** Katrien Houbrechts, Nicholas Marshall, Lesley Cockmartin, Hilde Bosmans

**Affiliations:** a KU Leuven, Department of Imaging and Pathology—Medical Physics and Quality Assessment, Leuven, Belgium; b UZ Leuven, Department of Radiology, Leuven, Belgium

**Keywords:** digital breast tomosynthesis, x-ray tube, flying focal spot, modulation transfer function, CDMAM

## Abstract

**Purpose:**

We characterize the flying focal spot (FFS) technology in digital breast tomosynthesis (DBT), designed to overcome source motion blurring.

**Approach:**

A wide-angle DBT system with continuous gantry and focus motion (“uncompensated focus”) and a system with FFS were compared for image sharpness and lesion detectability. The modulation transfer function (MTF) was assessed as a function of height in the projections and reconstructed images, along with lesion detectability using the contrast detail phantom for mammography (CDMAM) and the L1 phantom.

**Results:**

For the uncompensated focus system, the spatial frequency for 25% MTF value ($f_{25\%}$) measured at 2, 4, and 6 cm in DBT projections fell by 35%, 49%, and 59%, respectively in the tube-travel direction compared with the FFS system. There was no significant difference in $f_{25\%}$ for the front-back and tube-travel directions for the FFS unit. The in-plane MTF in the tube-travel direction also improved with the FFS technology.

The threshold gold thickness ($T_{t}$) for the 0.16-mm diameter discs of contrast detail phantom for mammography (CDMAM) improved for the FFS system in DBT mode, especially at greater heights above the table; $T_{t}$ at 45 and 65 mm improved by 16% and 24%, respectively, compared with the uncompensated focus system. In addition, improvements in calcification and mass detection in a structured background were observed for DBT and synthetic mammography. The FFS system demonstrated faster scan times (4.8 s versus 21.7 s), potentially reducing patient motion artifacts.

**Conclusions:**

The FFS technology offers isotropic resolution, improved small detail detectability, and faster scan times in DBT mode compared with the traditional continuous gantry and focus motion approach.

## 1 Purpose

Even though digital breast tomosynthesis (DBT) is a well-established imaging modality, efforts to optimize system accuracy and efficiency are still ongoing. Currently, different modes of gantry and/or detector movement exist. The two main acquisition modes for acquiring DBT projection data are a continuous gantry motion mode and a step-and-shoot mode. In the continuous mode, the x-ray tube moves continuously while x-rays are generated in short pulses at specific angles around the breast. This has the potential for faster acquisition times compared with the step-and-shoot operation but incurs source motion blurring along the direction of the source motion. Source motion blurring has been shown to reduce technical image quality, as quantified by a number of metrics.^1^^–^^5^ In a step-and-shoot mode, the x-ray tube stops at each angular location to acquire a projection image. This eliminates source motion blur provided that the tube stops without inducing any mechanical instability but may result in a longer scan acquisition time and therefore an increased chance for patient motion artifacts.^6^

It is likely that a wide-angle DBT system using a step-and-shoot approach to eliminate source motion blur would result in unacceptably long acquisition times. This work investigates the technical performance of a novel x-ray tube technology with a “flying focal spot” (FFS). Although the x-ray tube with the anode target still moves continuously during the DBT scan, the electron beam moves in the opposite direction compared with the tube movement during the x-ray pulse. The term “flying” therefore refers to the opposite movement of the electron beam, which in fact results in a focus that remains stationary for each projection. This technology is expected to reduce or eliminate source motion blurring while allowing for reduced scan acquisition time.^7^

This study compares two wide-angle DBT systems with two different acquisition modes: the continuous mode and the new FFS technology with a stationary focus during image acquisition. The impact on image sharpness and visibility of calcification- and mass-like test objects is quantified using technical image quality metrics.

## 2 Methods

Two DBT systems were investigated in this study: a Siemens MAMMOMAT Revelation and a Siemens MAMMOMAT B.brilliant. Both systems acquire 25 projections over an angular range of 50 deg. The Siemens MAMMOMAT Revelation employs the continuous motion acquisition mode, which incurs blurring due to focus motion during each projection.^8^^,^^9^ Hereafter, this is referred to as the “uncompensated focus” system. On the other hand, the Siemens MAMMOMAT B.brilliant is equipped with an x-ray tube with an FFS to mitigate source motion blurring in the acquired projection images. The FFS is utilized exclusively during acquisitions involving gantry movement, where compensation for source motion is needed. Therefore, in the digital mammography (DM) mode, both systems operate with a static focus, i.e., the FFS mechanism is not active when the gantry is not rotating. The technical specifications of both systems can be found in Table 1. In addition, the B.brilliant system incorporates a next-generation DBT reconstruction algorithm.^7^ The algorithms of both systems are based on a filtered back projection algorithm with non-linear iterative optimization steps.

**Table 1:** System specification comparison of two wide-angle DBT systems: one with an x-ray tube with moving focus during image acquisition (Siemens MAMMOMAT Revelation) and one with an x-ray tube with FFS technology (Siemens MAMMOMAT B.brilliant).

System name	MAMMOMAT Revelation “Uncompensated focus system”	MAMMOMAT B.brilliant “FFS system”
X-ray tube		
Anode target	Tungsten	Tungsten
Filter	50 μm Rh (DM)	1000 μm Al (DM)
	50 μm Rh (DBT)	700 μm Al (DBT)
Tube motion	Continuous tube motion	Continuous tube motion with FFS technology
Detector		
Material	Amorphous selenium	Amorphous selenium
Pixel size	85 μm×85 μm	85 μm×85 μm
DBT acquisition		
Angular range	±25 deg	±25 deg
Number of projections	25	25
Reconstruction algorithm	EMPIRE	PREMIA

### 2.1 X-ray Tube and Characterization of Geometric Blurring

#### 2.1.1 Focal spot dimensions

The focal spot dimensions were measured in DM and DBT modes with a multi-pinhole test object attached at the tube exit. This test object comprised 50-μm diameter holes, arranged in a 5×3 grid, in a 0.2-mm-thick brass plate. The system parameters were manually set to values that were closest to the automatic exposure control (AEC) settings for a 40-mm polymethyl methacrylate (PMMA) slab. Five DM “For Processing” images were acquired at 28 kV and 63 mAs on the Revelation, and at 26 kV and 90 mAs on the B.brilliant. Next, DBT projection images were acquired in a 0-deg angle stationary mode on both systems at 28 kV and 125 mAs, and 27 kV and 90 mAs. The focal spot dimensions in the front-back and tube-travel directions were estimated at 50 mm from the chest-wall edge and left-right centered. The focus sizes were measured between the points where the normalized profiles fell to 15% for both axes. The sizes were measured in the first projection of the 0-deg angle stationary DBT mode to reduce the potential influence of lag.

#### 2.1.2 MTF in projections

The presampling modulation transfer function (MTF) was measured on both systems in DM and DBT imaging modes using a version of the angled edge method.^10^ Clinically relevant acquisition parameters were established in the AEC mode by imaging PMMA blocks to simulate breast equivalent thicknesses from 2.1 to 7.5 cm.^11^ A 0.8-mm-thick stainless steel edge was then positioned at heights of 2, 4, and 6 cm above the breast support table. Acquisition parameters were manually set to the parameters used for the breast thickness relevant to the edge height above the table. An additional aluminum filter of 2-mm thick was added at the tube exit, and five DBT scans were acquired. The MTF in the central DICOM “For Processing” projection image was measured for the tube-travel and front-back directions. The measured MTF includes both detector and geometric blurring due to the x-ray focal spot. In addition, MTF measurements were performed in 0-deg angle stationary DBT mode using the same system parameters to obtain a baseline MTF without source motion blurring. To characterize x-ray detector sharpness in DM mode, the presampling MTF was also measured with the edge positioned on the breast support table. For all MTF measurements, monotonic conditioning was applied to the edge spread function (ESF), and the spatial frequency corresponding to the 25% MTF value ($f_{25\%}$) was determined.

To investigate the stability of the FFS over an extended period of time, MTF was measured every weekday for one month. The stainless steel edge was positioned on a 40-mm PMMA block, and a DBT acquisition was acquired in manual mode with system parameters closest to the clinical AEC settings for a 40-mm PMMA slab. The MTF was calculated for the tube-travel and front-back directions in the central DICOM “For Processing” projection, and $f_{25\%}$ was determined.

#### 2.1.3 MTF in reconstructed planes

To examine the in-plane MTF in the DBT reconstructed planes, a 25-μm diameter tungsten wire stretched between two PMMA plates, each 5-mm thick, was used. The wire phantom was positioned on top of 15-, 35-, and 55-mm PMMA, oriented in both the front-back and tube-travel directions at an angle of 1 to 3 deg relative to the detector pixels. For each height, five DBT images were acquired on both systems with system parameters adjusted to match the clinical AEC settings for these equivalent breast thicknesses. Due to the combination of a low signal from the wire running parallel to the source motion direction and high levels of image noise, this work only presents the in-plane MTF in the tube-travel direction as a function of height. As the B.brilliant system has advanced artifact reduction processing for attenuating objects that produce image signal levels above a certain threshold,^7^ erroneous in-plane MTF results were obtained for this system when the wire was positioned in the air. This prompted the imaging of the wire on PMMA as a means of reducing signal contrast from the wire and suppressing the influence of the artifact reduction algorithm. Monotonic conditioning was not applied to the oversampled line spread function (LSF) generated from the wire image as this removed some features present in the LSF.

#### 2.1.4 DBT scan time

The total scan time of a DBT acquisition was measured on both systems using a Piranha dosimeter (RTI Electronics AB, Sweden). The acquisition parameters were again manually set to the clinical AEC settings for a 40-mm PMMA slab. No pre-shot was included in this time measurement.

### 2.2 CDMAM as a Function of Height

To investigate the impact of either uncompensated focus or FFS on technical image quality, a contrast detail phantom for mammography (CDMAM 3.4) (Artinis, Netherlands) was used to measure small detail detectability. This phantom contains small gold disks with detection thresholds that have been shown to correlate with microcalcification detectability in DM.^12^ The CDMAM phantom was positioned on top of 1-cm PMMA, and subsequently, PMMA slabs were added to obtain a total of six different phantom thicknesses simulating breast equivalent thicknesses of 2.1, 3.2, 4.5, 6, 7.5, and 9 cm according to Dance et al.^13^ The PMMA was added between the breast support table and the CDMAM phantom, resulting in CDMAM being imaged at heights of 15, 25, 35, 45, 55, and 65 mm above the table. Acquisition parameters were manually set to relevant settings established in AEC mode for these six breast thicknesses. PMMA wedges, ∼2-mm thick, were placed under the PMMA phantoms to compensate for the slight slope of the table and to ensure that the CDMAM phantom was positioned parallel to the detector. For each height, eight images were acquired in both DM and DBT modes, for both systems. The Revelation system and B.brilliant system utilize different reconstruction algorithms, optimized for the relevant system hardware and expected geometric blurring. Automatic read-out of the CDMAM images was performed using CDCOM (DM: v1.6, DBT: v1.5.2) with established processing steps^14^ to obtain the threshold gold thickness ($T_{t}$). As both x-ray tubes have different filtrations, the AEC mode did not result in the same mean glandular dose (MGD). Therefore, the following equation was used to scale the threshold gold thicknesses acquired at ${\mathrm{MGD}}_{\mathrm{FFS}}$ on the system with FFS ($T_{{\mathrm{FFS}\;\text{at}\;\mathrm{MGD}}_{\mathrm{FFS}}}$) to a similar MGD as the system with the uncompensated focus ($T_{{\mathrm{FFS}\;\text{at}\;\mathrm{MGD}}_{\text{uncompensated focus}}}$):

$$T_{{\mathrm{FFS}\;\text{at}\;\mathrm{MGD}}_{\text{uncompensated focus}}}=T_{{\mathrm{FFS}\;\text{at}\;\mathrm{MGD}}_{\mathrm{FFS}}}\cdot \sqrt{\frac{{\mathrm{MGD}}_{\mathrm{FFS}}}{{\mathrm{MGD}}_{\text{uncompensated focus}}}.}$$ (1)

This assumes that quantum noise is the dominant source of noise in the DM projection images and the DBT reconstructed images.

### 2.3 L1 Phantom as a Function of Height

The L1 phantom^15^ comprises a breast-shaped PMMA container filled with water and PMMA spheres of different diameters. Within the phantom are 3D-printed mass lesions, both non-spiculated and spiculated, ranging in size. The non-spiculated masses have average diameters of 1.5, 2.1, 3.0, and 4.3 mm, whereas the spiculated lesions are 4.4-, 6.1-, 8.8-, and 12.2-mm large. In addition, calcium carbonate (${\mathrm{CaCO}}_{3}$) particles, representing microcalcifications, are arranged in five clusters, each containing five microcalcifications with diameters in the range of 90 to 100, 112 to 125, 140 to 160, 180 to 200, and 224 to 250  μm. The calcifications in the L1 phantom have recently been replaced compared with the original phantom; a more accurate size measurement of the individual calcifications was applied before their inclusion in the phantom. The phantom has a physical thickness of 48 mm and an approximate breast equivalent thickness of 60 mm. The lesions are located at a height of 24 mm.

Initially, the L1 phantom was positioned on the breast support table of both systems, and spacers were used to set the compressed thickness to 60 mm. DM and DBT images were then acquired in the AEC mode to determine the exposure settings for the L1 phantom on both systems summarized in Table 2. Next, 12 DM images and 12 DBT scans of the L1 phantom without spacers were acquired on both systems using the established AEC settings. To investigate the detectability performance of both systems as a function of height above the breast support table, the lesion height was further increased to 54 mm by placing 3-cm styrofoam spacers underneath the L1 phantom. The acquisitions were repeated using the same exposure settings. The MGD was calculated using the method of Dance et al.^11^ (Table 1). Between each acquisition, the L1 phantom was shaken to create unique background patterns.

**Table 2:** DM and DBT acquisition parameters for the L1 phantom together with the calculated MGD.

	MAMMOMAT Revelation				MAMMOMAT B.brilliant			
	Anode/filter	kV	mAs	MGD (mGy)	Anode/filter	kV	mAs	MGD (mGy)
**DM**	W/Rh (50 μm)	30	100	1.15	W/Al (1 mm)	27	140	1.30
**DBT**	W/Rh (50 μm)	30	200	2.15	W/Al (0.7 mm)	28	140	2.30

For the human observer study, regions of interest of 20  mm×20  mm were extracted from DM images and synthetic mammograms (SM) and volumes of interest of 20  mm×20  mm×9  mm from DBT reconstructions. For each system, phantom height setup, and imaging modality, 12 signal-present samples were obtained with the relevant lesion positioned centrally along with 200 signal-absent background samples. Subsequently, a four-alternative forced choice study was set up with dedicated software^16^ presenting in each reading session 12 image sets (one signal-present and three signal-absent samples). Six medical physicists performed this signal/location-known-exactly and background-known-statistically detection task, after appropriate training. The percentage of correctly detected targets [percentage correct (PC)] was calculated for each reading session and reader separately. A logistic regression analysis was used to investigate the impact of the imaging system, lesion height, and imaging modality on target detection. To evaluate the consistency of PC values among different observers, the intraclass correlation coefficient (ICC) was calculated.

## 3 Results

### 3.1 X-ray Tube and Characterization of Geometric Blurring

**Fig. 1:**
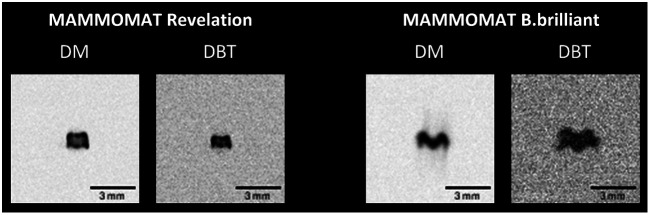
DM and DBT “For Processing” image of the focal spot of a MAMMOMAT Revelation and a MAMMOMAT B.brilliant, acquired with a multi-pinhole test object. DBT images were acquired in 0-deg angle stationary mode. The horizontal axis is the front-back direction, and the vertical axis is the tube-travel direction.

The focus size results are given as (length in the tube-travel direction) × (length in the front-back direction). In the DM mode, the focus sizes for the Revelation were (0.39±0.01)×(0.50±0.01)  mm, whereas focus sizes for the B.brilliant were (0.27±0.01)×(0.56±0.01)  mm. Similar focus size results were observed in the DBT mode for the Revelation, with values of (0.33±0.01)×(0.47±0.01)  mm. Slightly larger dimensions were measured for the B.brilliant in DBT mode: (0.41±0.02)×(0.79±0.02)  mm. The DBT focus size measurements do not include source motion blur as they were obtained in 0-deg angle stationary mode. For both systems, the largest focus dimension was in the front-back direction, as can also be seen in the focus images in Fig. 1. Note that some degree of dose dependency is expected for the measured focus size.

**Fig. 2:**
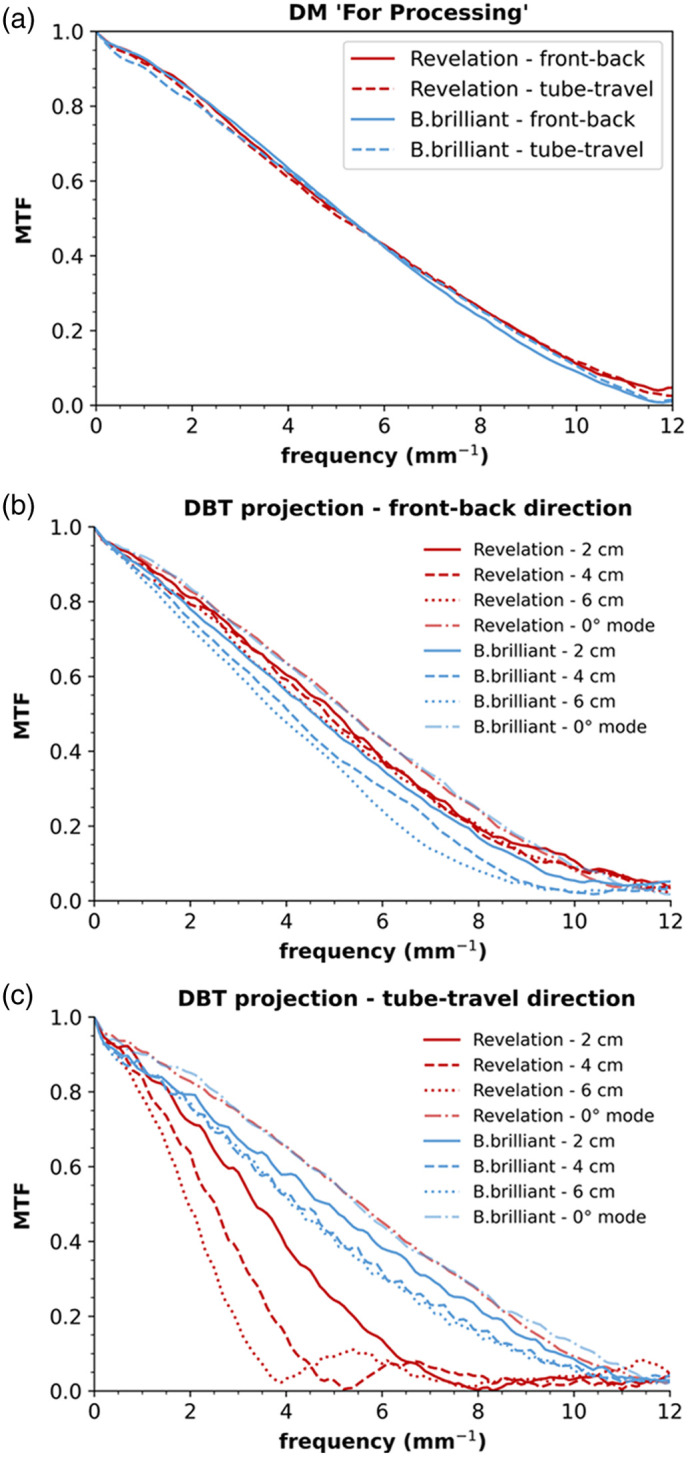
Average MTF in front-back and tube-travel direction obtained from five DICOM “For Processing” DM images (a) and five central DBT projection images (b), (c) acquired on a MAMMOMAT Revelation and a MAMMOMAT B.brilliant. The dash-dotted curves were obtained at 2-cm height in 0-deg angle stationary DBT mode, excluding source motion blur. MTF values at 4 and 6 cm in 0-deg mode, not shown, were similar to those at 2 cm.

**Fig. 3:**
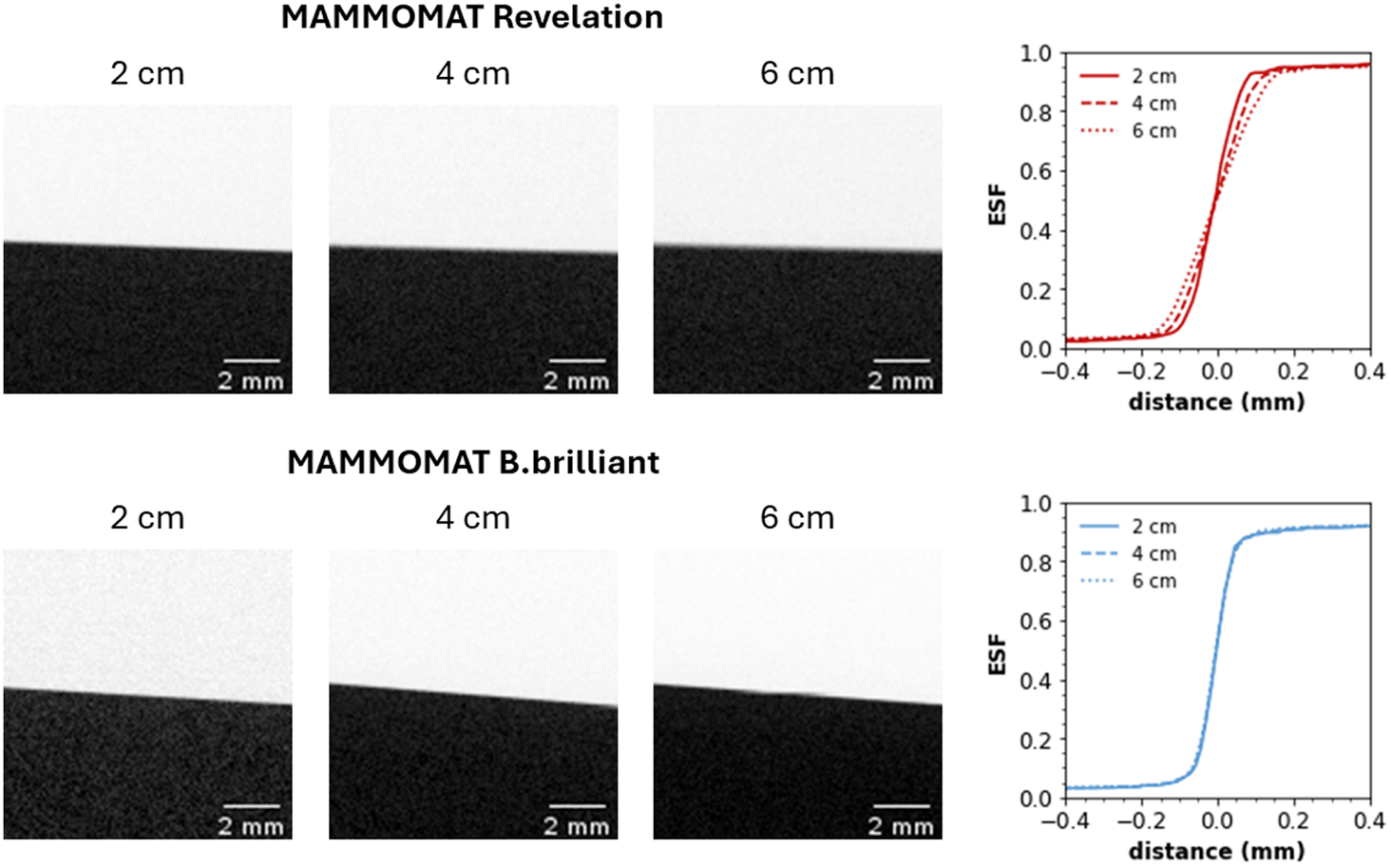
Central DBT projection of MTF edge in the tube-travel direction, together with their ESF measured at 2, 4, and 6 cm above the table using a MAMMOMAT Revelation and a MAMMOMAT B.brilliant.

Figure 2(a) shows that the DM MTF curves are similar for both systems. There is very little influence of focus size on the presampling MTF in DM mode as the edge was positioned on the breast support table, minimizing geometric blurring. Figure 2(b) shows the MTF curves measured in DBT projections in the front-back direction. The MTF gradually decreases with increasing height above the table due to focus size geometric blurring. The MTF for the B.brilliant system with the FFS is slightly lower compared with the Revelation system, consistent with the larger focus observed for this system, especially in the front-back direction. In the tube-travel direction, there is an additional blurring due to source motion for the Revelation, which results in a significant decrease in MTF as a function of height [Fig. 2(c)]. This may also be present for the B.brilliant with FFS if the x-ray tube motion is not entirely canceled by the electromagnetic deflection of the focus; however, this was not seen in these measurements. Figure 3 shows an image of the edge in the central DBT projection for the two systems, along with a graph of the ESF in which some broadening of the edge transition can be seen for the Revelation compared with the B.brilliant.

For B.brilliant with the FFS in DBT mode, $f_{25\%}$ at 2, 4, and 6 cm above the table was respectively 7.1±0.2, 6.7±0.1, and $6.0\pm 0.1\;{\mathrm{mm}}^{-1}$ (front-back) and 7.6±0.2, 6.8±0.1, and $6.6\pm 0.1\;{\mathrm{mm}}^{-1}$ (tube-travel). The difference in $f_{25\%}$ in the front-back and tube-travel direction is smaller for B.brilliant compared with Revelation, resulting in an improved MTF isotropy for B.brilliant. The FFS tube in the B.brilliant gives notably higher $f_{25\%}$ values in the tube-travel direction compared with the Revelation system. The $f_{25\%}$ values at 2, 4, and 6 cm above the table were respectively 35%, 49%, and 59% higher.

**Fig. 4:**
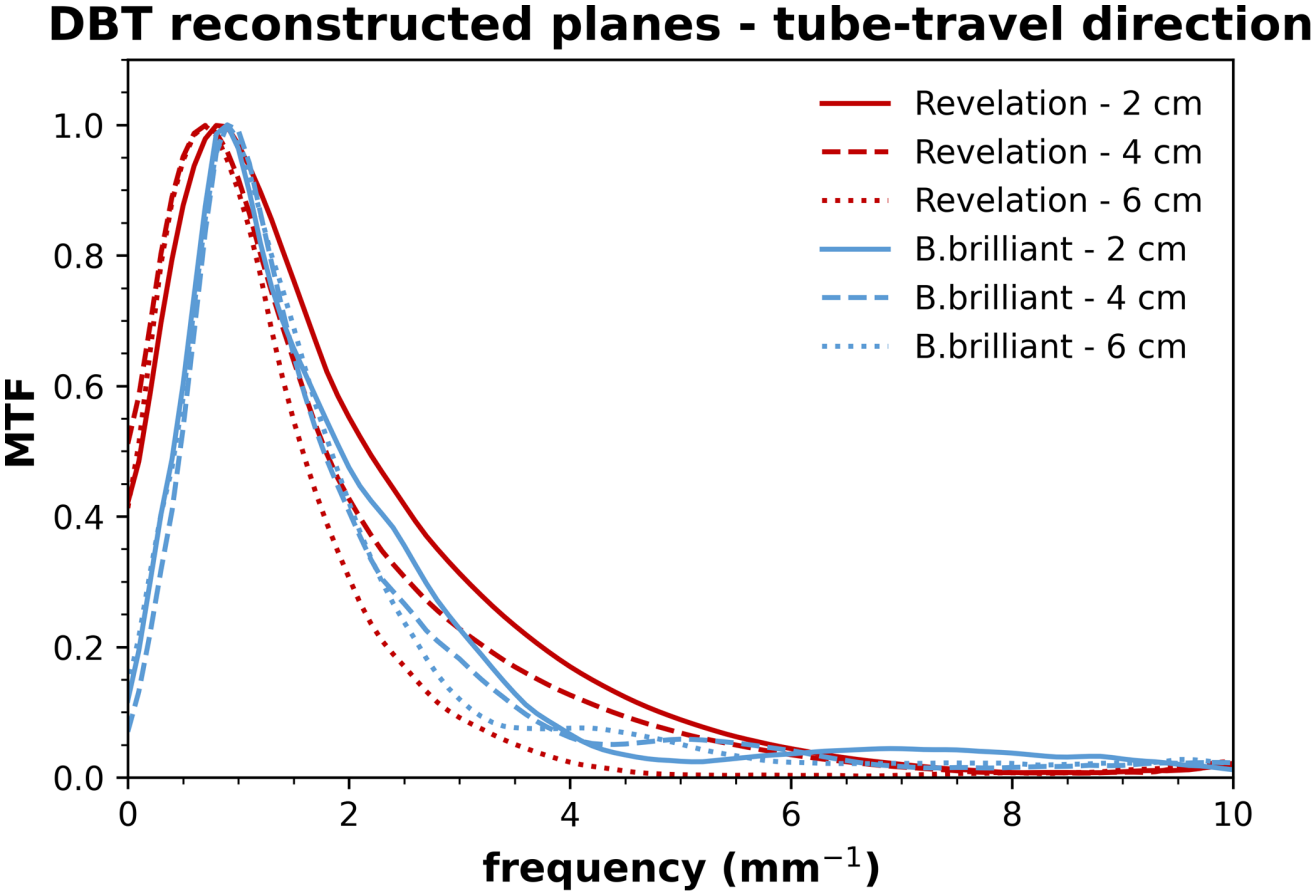
In-plane MTF measured in the tube-travel direction at different heights in reconstructed DBT slices of a MAMMOMAT Revelation and a MAMMOMAT B.brilliant.

**Fig. 5:**
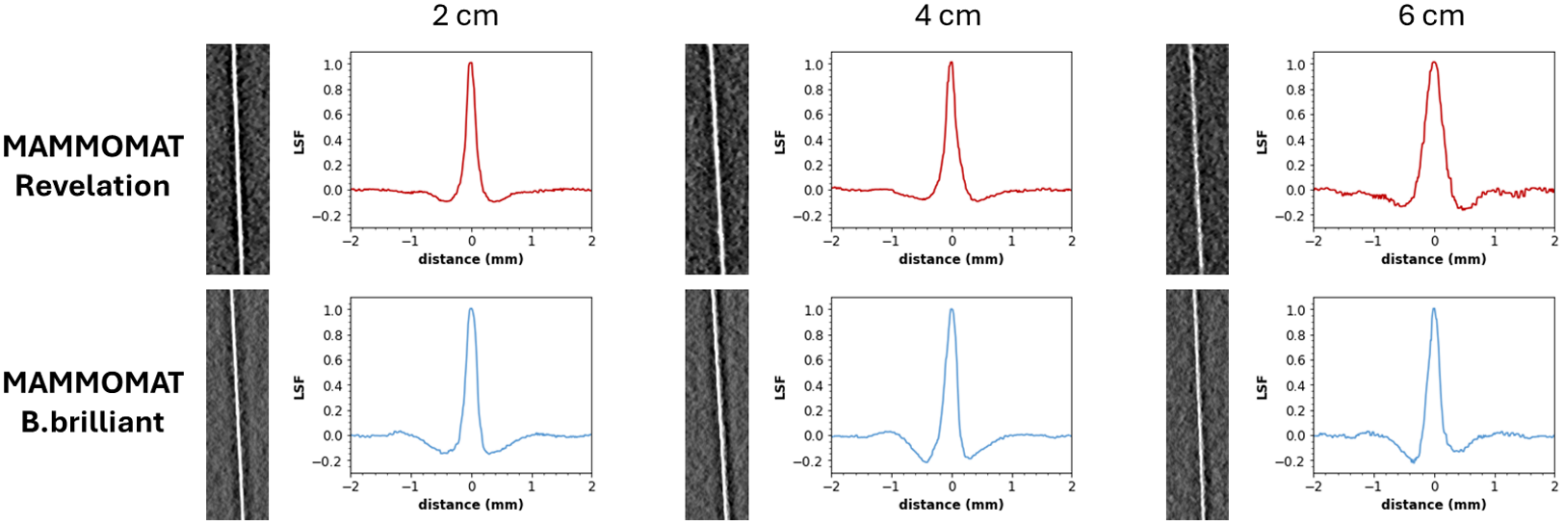
In-focus reconstructed DBT slice of tungsten wire running perpendicular to the chest wall edge imaged on top of 2, 4, and 6 cm PMMA, together with the LSF. Both LSFs have some overshoot in the tails, which is more pronounced in the B.brilliant data, causing a reduction in MTF at spatial frequencies below $∼1\;{\mathrm{mm}}^{-1}$.

The MTF in DBT reconstructed planes, i.e., the in-plane MTF, gives information on the blurring present in the DBT projection images but also includes the influence of image reconstruction on image sharpness. A different reconstruction algorithm is implemented on both systems, with each algorithm being optimized for the acquisition geometries of its respective system. The in-plane MTF measured in the tube-travel direction at different heights in the reconstructed DBT planes and normalized to the curve peak is shown in Fig. 4. The corresponding LSFs are shown in Fig. 5. The in-plane MTF in the tube-travel direction deteriorates with increasing height. The degradation is most pronounced for the Revelation system, which shows a 41% reduction in $f_{25\%}$ from 2 to 6 cm, compared with a 12% reduction for the B.brilliant system. The drop in MTF in the low spatial frequency region ($<1\;{\mathrm{mm}}^{-1}$) for both systems is probably due to the application of a ramp filter in the reconstruction.

**Fig. 6:**
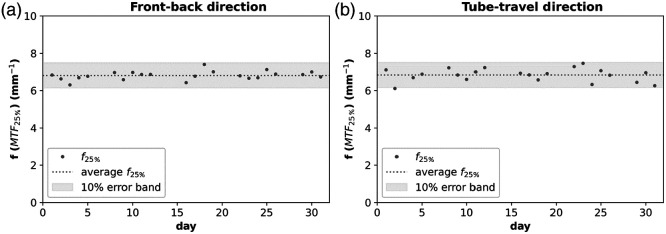
Spatial frequency of the 25% MTF value in the front-back (a) and tube-travel (b) direction measured in DBT projections of the MAMMOMAT B.brilliant over one month.

Figure 6 shows the spatial frequency where the MTF drops to 25% ($f_{25\%}$) measured in DBT projections as a function of time for the B.brilliant system with FFS. The deviations are within 10% of the average $f_{25\%}$ value over a month of measurements. This demonstrates that the FFS technology is stable over time.

**Fig. 7:**
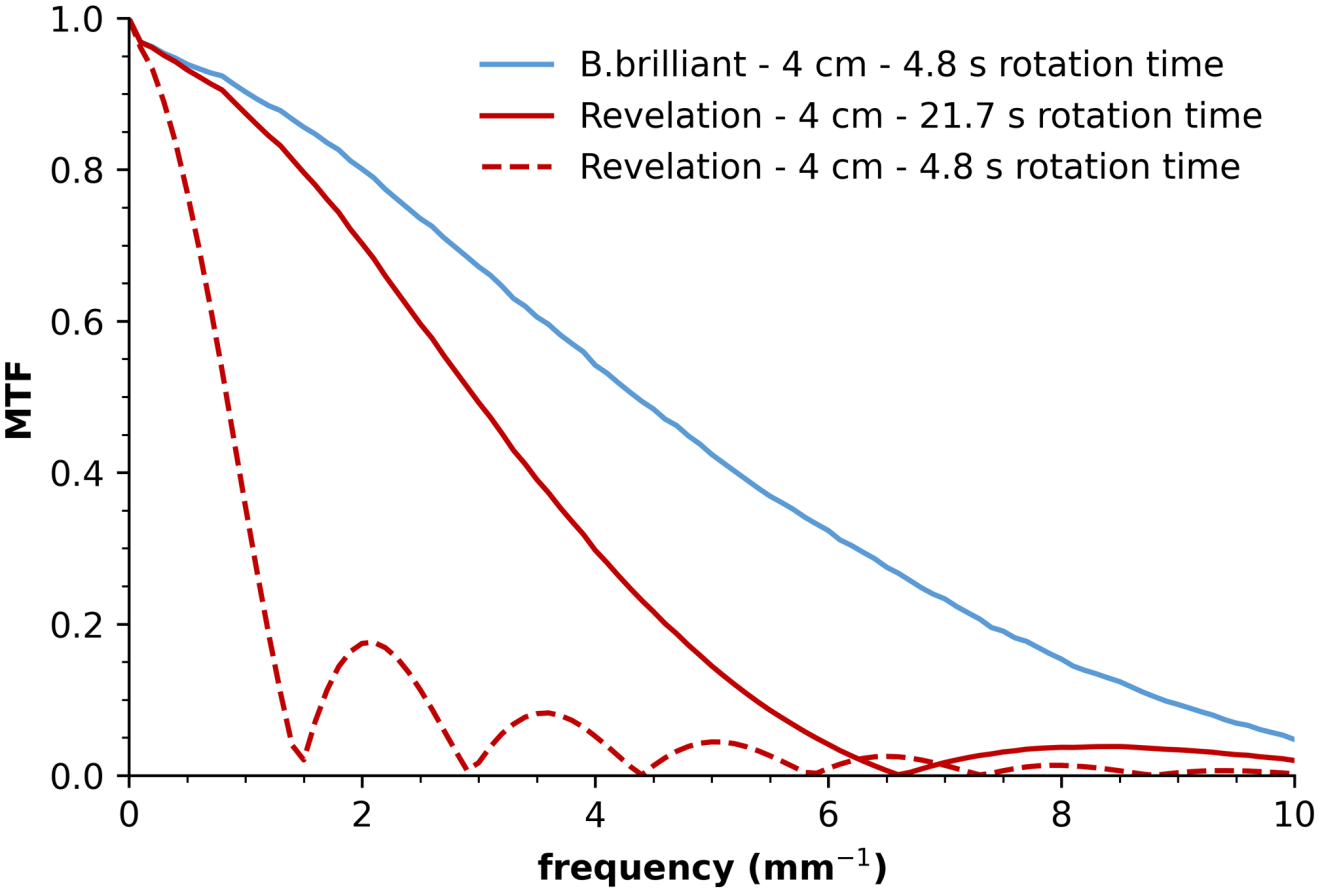
Analytically modeled DBT projection MTF in the tube-travel direction at 4 cm above the breast support table for the B.brilliant system with clinical rotation time (4.8 s), the Revelation system with clinical rotation time (21.7 s), and the Revelation system if it would operate with 4.8-s rotation time.

A DBT acquisition on the Revelation system requires ∼21.7  s, whereas this is reduced to 4.8 s for the B.brilliant system. This results in a gantry speed of 2.3  deg/s for the Revelation compared with 10.4  deg/s for the B.brilliant. If the gantry speed of the Revelation would be increased to 10.4  deg/s, without compensating for source motion, then the focus size due to motion would rise from 1.9 to 8.8 mm. To evaluate the impact of focus motion, the DBT projection MTF was modeled as the product of the presampling MTF and a sinc function describing the projected focus length due to motion.^8^^,^^9^ As a result, $f_{25\%}$ for the DBT projection MTF in the tube-travel direction at 40 mm above the breast support table would theoretically decrease from 4.3 to $1.1\;{\mathrm{mm}}^{-1}$ (Fig. 7), resulting in unacceptable blurring of the projections. The x-ray tube filtration has been changed from Rh to Al in the B.brilliant, which will allow higher tube outputs, enabling an increased acquisition rate for the same total scan dose.^17^ This significant reduction in acquisition time has the potential to reduce patient motion artifacts.

### 3.2 CDMAM as a Function of Height

**Fig. 8:**
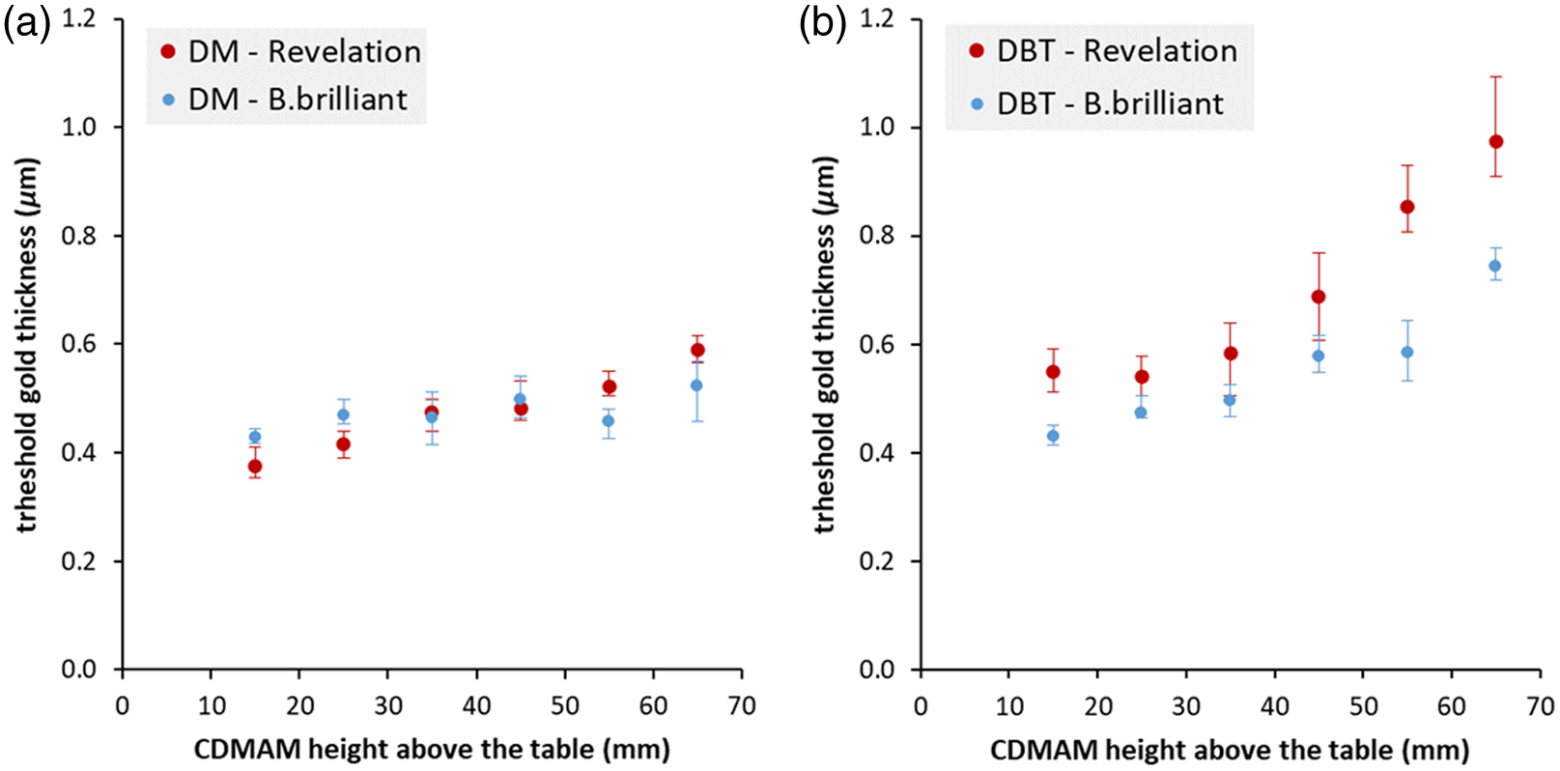
Threshold gold thickness for the 0.16 mm discs retrieved from the CDMAM phantom imaged in DM (a) and DBT (b) mode on a MAMMOMAT Revelation and MAMMOMAT B.brilliant. Threshold gold thicknesses were corrected for MGD. Error bars indicate the 95% confidence interval.

Figure 8 shows the threshold gold thickness for the 0.16-mm diameter discs of the CDMAM phantom positioned at different heights above the table. The results of the 0.16-mm discs are presented to avoid any extrapolation for the reported threshold gold thicknesses. Nonetheless, similar results were observed for smaller-diameter CDMAM discs.^18^

From system design parameters, we might expect some differences in the DM results between the systems. Although both systems use the same model of x-ray detector and anti-scatter grid, and a static focus, the B.brilliant has an Al filter compared with the Rh filter in the Revelation. The initial contrasts of the gold discs in the phantom are higher for the Rh-filtered spectrum, which might give some improvement in threshold gold thickness performance. However, in the DM mode, similar threshold values are seen for the two devices, once adjusted for dose. For both systems, the detection threshold increases with height above the table as a result of geometric blurring by the extended focal spot.

**Fig. 9:**
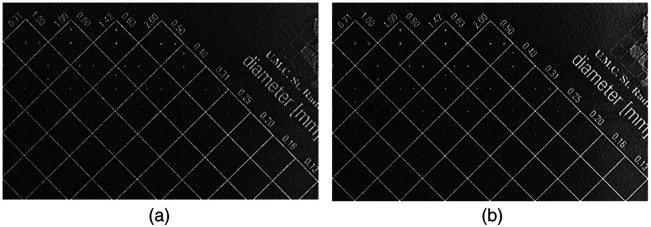
In-focus reconstructed DBT slice of the CDMAM phantom positioned 65 mm above the table, acquired on a MAMMOMAT Revelation (a) and a MAMMOMAT B.brilliant (b). The images display variations in window-leveling due to different x-ray spectra and reconstruction algorithms used by the systems.

In the DBT mode, the detection thresholds improved for the system with FFS, particularly at greater heights above the table. The $T_{t}$ values at 25, 45, and 65 mm above the table improved by 12%, 16%, and 24%, respectively, for the B.brilliant compared with the Revelation. These results are generally consistent with the measured in-plane MTF curves. However, the CDMAM results will also be affected by the anode/filter combination, as in the DM mode, and by the reconstruction algorithm. Figure 9 shows the in-focus reconstructed DBT slice of the CDMAM phantom at 65 mm above the table for both systems.

### 3.3 L1 Phantom as a Function of Height

**Fig. 10:**
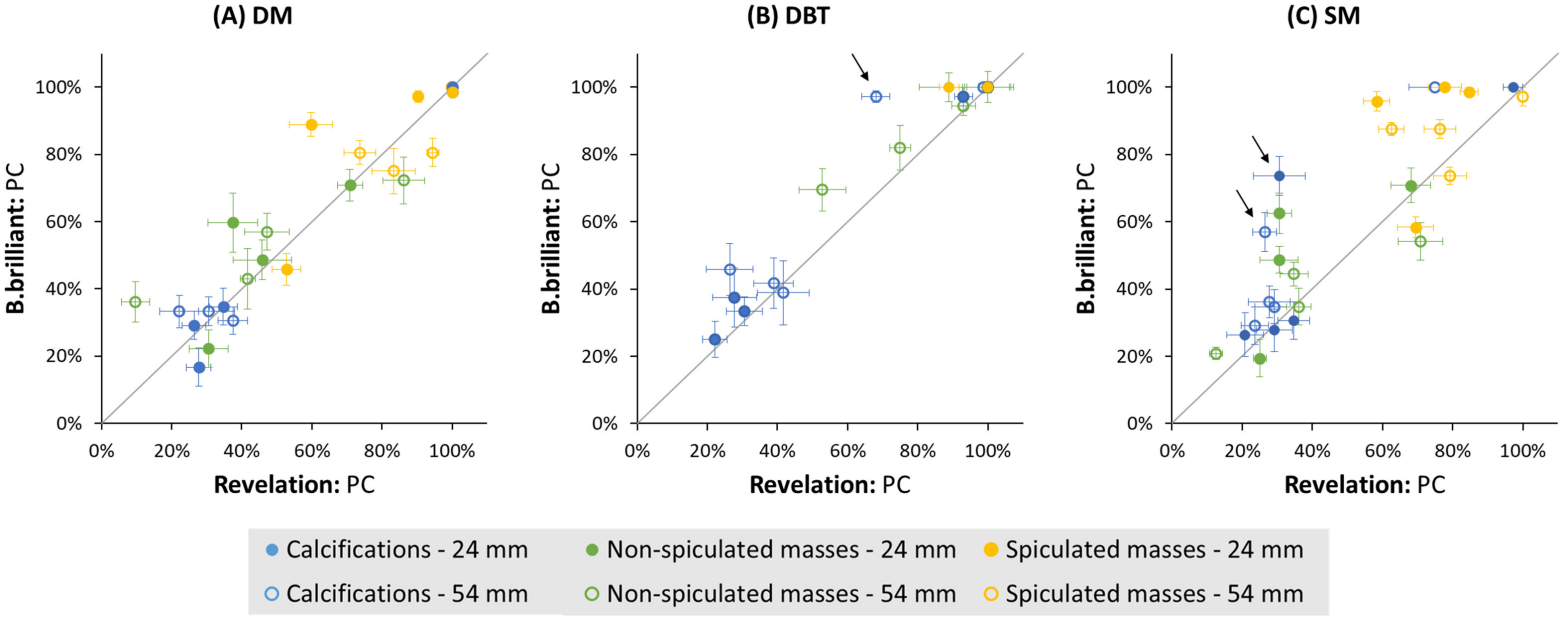
Reader-averaged percentage of correctly detected targets (PC) in L1 images acquired on a MAMMOMAT B.brilliant against images acquired on a MAMMOMAT Revelation. Error bars represent the standard error of the mean.

The reader-averaged percentage correct (PC) values of each lesion in the L1 phantom are presented in Fig. 10 for all three image types, for the two systems. A substantial inter-reader agreement was found (ICC = 0.77). In DM, no performance difference in detecting calcifications within the L1 structured background was observed between the two systems (p=0.73), irrespective of lesion height. These results are consistent with the CDMAM results, even though detection was here studied in processed DM images. The B.brilliant system demonstrated a significantly improved detection of non-spiculated masses (p=0.012), but only a slight, non-significant improvement for spiculated masses.

**Fig. 11:**
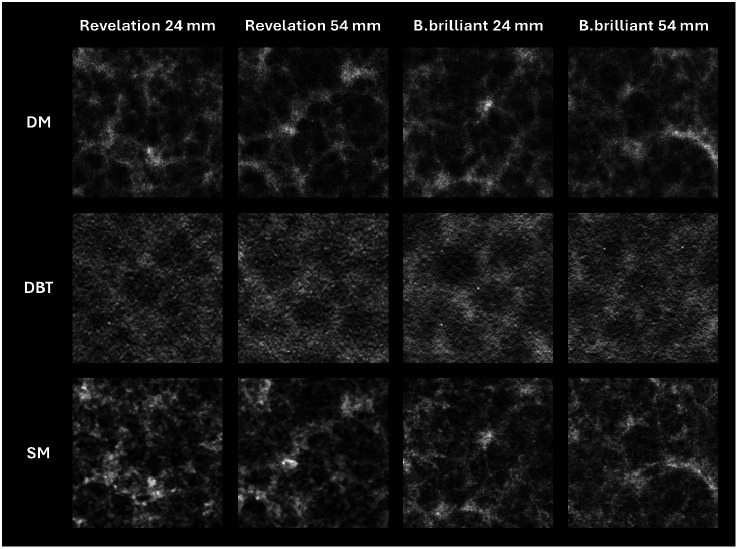
Visualization of a calcification cluster in the L1 phantom with three different modalities using a MAMMOMAT Revelation and a MAMMOMAT B.brilliant. The cluster consists of five calcifications, each 180 to 200  μm in diameter, arranged in a dice pattern and positioned at heights of 24 and 54 mm above the table.

Overall, the B.brilliant showed better detection of all three lesion types in DBT images compared with the Revelation (p<0.0001). The odds of detecting calcifications were 1.56 times higher with B.brilliant compared with Revelation (95% CI [1.21, 2.02]; p=0.001). For the 180 to 200  μm calcification cluster located 54 mm above the table, the PC dropped by almost 30% for Revelation compared with a PC of 97% for B.brilliant [see arrow Fig. 10(b)]. This difference can also be observed in Fig. 11. However, no significant impact of calcification height was observed (p=0.37) contrary to what was found with CDMAM.

Some improvement was also seen in the SM images of B.brilliant compared with Revelation, showing significantly improved detection of calcifications and spiculated masses. The odds of detecting a calcification cluster in the L1 phantom were 1.80 times higher for B.brilliant compared with Revelation (95% CI [1.42, 2.27]; p<0.0001). For example, the PC of the 180- to 200-μm calcification cluster at 24- and 54-mm height was 43% and 31% higher, respectively, for the B.brilliant system [see arrows Fig. 10(c)]. Again, no significant effect of calcification height was observed (p=0.07). When comparing the three imaging modalities, the odds ratio for lesion detection remains higher in both DM (Revelation: OR = 1.72, 95% CI [1.49, 1.98]; B.brilliant: OR = 1.20, 95% CI [1.03, 1.39]) and DBT (Revelation: OR = 3.47, 95% CI [2.98, 4.04]; B.brilliant: OR = 3.48, 95% CI [2.96, 4.10]) compared with SM (p<0.0001).

## 4 Discussion

The FFS technology in the Siemens MAMMOMAT B.brilliant system results in an improved MTF isotropy in DBT projections compared with the Siemens MAMMOMAT Revelation, which suffers from source motion blur. The MTF isotropy is now comparable to DBT systems that use the step-and-shoot approach.^19^ This suggests that the implementation of the flying focus adequately compensates for x-ray tube motion during the acquisition of each projection image, as the gantry moves around the breast in a DBT scan. Moreover, it has been demonstrated that this compensation remains consistent over a period of one month.

The FFS tube has a slightly larger focus area in the DBT mode, and this had some impact on the MTF in the front-back direction measured in the projections for B.brilliant versus Revelation. Nevertheless, the B.brilliant offers superior resolution in the tube-travel direction compared with Revelation. Zheng et al.^4^ demonstrated that a typical focal spot size up to ∼0.3  mm does not impact the reconstructed image resolution if the x-ray tube remains stationary during data acquisition. Conversely, source motion blur significantly degrades the reconstructed image resolution as also observed in this study.

Although there is a definite improvement in the in-plane MTF curve at 6 cm above the table for B.brilliant in the tube-travel direction, in-plane MTF results at 2 and 4 cm are similar for the two systems. The increase in the projection MTF for the B.brilliant is not seen in the in-plane MTF results for these heights, and this may be due to differences in processing. The MTF measured in-plane clearly includes the influence of spatial frequency filtering performed in the reconstruction algorithm.^20^ It is possible that the high in-plane MTF seen for the Revelation at 2 and 4 cm comes at the expense of amplified noise and the reduction seen at 6 cm comes from the strong geometric blurring at this height.

There are some limitations to the measurement of in-plane MTF using either a wire, edge, or disc^9^^,^^21^^,^^22^ and further depend on the test object used for the measurement. The presence of additional processing methods such as artifact reduction, which are object dependent, can also have a strong influence. Nevertheless, previous work^22^ on various DBT systems using different (including non-linear) reconstruction algorithms showed a reasonably good correlation between the detectability of small diameter discs and a Fourier-based detectability metric. In trying to explain the detectability of small details such as microcalcifications, the MTF cannot be used in isolation, and aspects such as signal profile and noise must also be considered.^23^ However, reducing geometric blurring in the acquired projection image dataset gives greater flexibility in the successive reconstruction and image processing steps for the system with the FFS tube.

Threshold gold thickness measured using the CDMAM test object in the DM images was lower for the Revelation system at 15 and 25 mm above the table, whereas slightly superior performance was seen for the B.brilliant at 65 mm. Overall, the FFS tube had no net impact on the technical image quality of the DM images. Spatial resolution, as quantified using MTF, and the detection of calcification- and mass-like objects in a structured background were similar in the DM mode for the two systems, despite differences in x-ray spectrum and resulting initial contrast, as well as differences in the image processing algorithms.

An improved small detail detectability in DBT mode was observed for the B.brilliant system. For the CDMAM phantom used in this study, notable improvements in the threshold gold thickness for 0.16-mm-diameter discs as well as smaller discs (not reported) were found, especially when the phantom was positioned at 55 mm or higher above the table. In addition to the increased, isotropic resolution, this may also be partly attributed to the reconstruction algorithm, which is known to influence lesion visibility.^24^ It should be noted that object detectability quantified using contrast-detail test objects depends on the signal-to-noise ratio (and therefore the dose level)^25^ and not just the resolution or sharpness of the detector and imaging system.^26^

The aim of this work was to compare the imaging performance with an FFS tube against that of an x-ray tube with an uncompensated focus. However, given the difference in anode/filter settings and processing algorithms, a like-for-like comparison was not possible. Manufacturer-determined AEC settings were used for imaging the test objects, and CDMAM results were corrected for differences in MGD to account for variations in quantum noise in the images. Nevertheless, some differences remain due to the difference in the x-ray spectrum.

In DM mode, the systems are set up to meet the image quality and dose requirements specified in the European DM protocol.^27^ After MGD correction, the CDMAM results in Fig. 8 show that only small differences exist between the two systems. For B.brilliant, the tube motion compensation is not activated in the DM mode, and therefore, any differences arising from the x-ray spectrum, scattered radiation, anti-scatter grid and x-ray detector are likely to be small.

In the DBT mode, the reconstruction algorithm will influence the threshold gold thickness results. The anode/filter combination may also slightly influence the results, although we would expect this effect to be similar or even smaller due to the thinner Al filter used in DBT compared with the DM mode in the B.brilliant. The MGD-corrected threshold gold thickness results are significantly lower for the B.brilliant in the DBT mode, consistent with the elimination of geometric blurring due to a compensated net focus motion relative to the breast during exposure.

The CDMAM phantom is designed to evaluate image quality in 2D mammography and must be used with caution when comparing performance among different DBT systems.^28^^,^^29^ The CDMAM results only provide information on the in-plane detectability of small details, without any influence from background structures and do not demonstrate the absolute performance of both DBT systems. Both systems have an angular range of 50 deg, and therefore, the influence of the tomography angle on the magnitude of in-plane structured noise will be very similar. There may however be differences due to the reconstruction algorithm, which can influence the artifact spread function and therefore the level of in-plane anatomical noise.^19^

PMMA was used to support the CDMAM phantom when assessing threshold detectability at various heights above the table. Although this allowed the use of clinically relevant technique factors for the different breast equivalent thicknesses, the use of PMMA increased the amount of scattered radiation present, especially for DBT acquisitions as there is no anti-scatter grid present in this mode. Scatter will reduce threshold detectability; however, the effect is expected to be similar for both systems. Both units use the same detector technology and the same anti-scatter grid in DM mode and no grid in DBT mode. The difference in x-ray beam energy between the systems only has a small influence on the scatter-to-primary ratio,^30^ and therefore, the impact of scatter on small detail detectability is likely comparable for both systems.

To support the CDMAM results, calcification detection was also assessed using a 3D phantom with a structured background. Improved lesion detection in DBT was again observed for the B.brilliant, although the effect of the height of the lesion above the breast support table was less pronounced than in the CDMAM results. One possible reason for this is the greater uncertainty associated with these results as they were obtained with human readers rather than automatic readout as used for CDMAM.^15^ The background structure clearly plays a role in the greater uncertainty of the measured detection thresholds. In addition, it was observed that the current calcification size groups in L1 were either visible at a 100% PC level or not visible at all; there were almost no calcification diameters with a 50% to 80% PC detection rate that exhibited subtle visibility. Smaller effects that impact detection, such as the height of the lesion, may not be detected with the current set of calcification diameters in the L1 phantom.

When lifting the L1 phantom above the table, we chose not to use PMMA supports, unlike the CDMAM phantom. Combining 30 mm of PMMA with the L1 phantom would result in a breast-equivalent thickness of ∼100  mm,^11^ which is not very common in clinical practice. Furthermore, using PMMA would alter the L1 phantom composition, change the structured noise generated by the phantom,^15^ and potentially change the difficulty of the detection task. For these reasons, we opted to use 30 mm of polystyrene instead. This choice ensures that lesion detectability results between the two phantom thicknesses are not influenced by changes in background structure and are therefore more likely to be related to differences in system performance parameters. The primary goal of this study is to compare the two systems using like-for-like phantom compositions whenever possible.

There was a difference in MGD between the systems when imaging the L1 phantom, which was difficult to correct because the effect of dose on the signal-to-noise ratio within a structured background does not follow the Rose model as is the case for the homogeneous CDMAM phantom.^31^ The AEC configuration of the B.brilliant gave a 13% higher MGD for the L1 phantom in DM compared with the Revelation and a 7% higher MGD for DBT. Based on the work of Vancoillie et al.,^31^ the impact of this small dose difference on detectability was considered negligible when comparing both systems.

The SM images of the L1 phantom demonstrated that the improved sharpness and isotropy in DBT mode for the B.brilliant system were exploited in the SM processing, resulting in the improved detection of calcifications and spiculated masses compared with the Revelation system. Nevertheless, the performance of SM has not yet reached the performance achieved in DM and DBT as seen in technical image quality evaluations for other DBT devices.^31^^,^^32^

The FFS x-ray tube in the B.brilliant system reduced the DBT scan acquisition time, from 21.7 s for the Revelation to 4.8 s for a 45-mm breast equivalent thickness. If this scan time would be implemented in the Revelation system without FFS technology, we could expect a drop of ∼70% in $f_{25\%}$ for the DBT projection MTF at 40 mm above the breast support table.^20^ Ghani et al.^3^ observed a decrease of ∼17% in cutoff frequency (10% MTF) of the in-plane MTF when doubling the gantry speed in continuous motion mode for a narrow-angle DBT system. The FFS technology allows wide-angle DBT scans to be performed with a scan time similar to that found in narrow-angle systems.^19^ This has clear potential to reduce patient motion artifacts, which will likely improve image quality.^33^ An additional benefit of the continuous gantry motion used with the FFS tube compared with a step-and-shoot method might include the elimination of small vibrations in the gantry and breast support platform as the gantry accelerates and decelerates during the scan.

## 5 Conclusion

The introduction of an FFS x-ray tube in the Siemens MAMMOMAT B.brilliant system brings established technology from CT imaging to DBT imaging systems. This has greatly improved MTF isotropy in DBT projections, effectively eliminating the relative motion of the source during exposure. As a result, scan speed has been increased by a factor of ∼4 without introducing source motion blur, an issue that would severely degrade image sharpness if the same gantry rotation speed was applied to the Siemens MAMMOMAT Revelation. Compared with the Revelation, which has a moving (uncompensated) focus during exposure, the B.brilliant system with FFS tube demonstrated superior resolution in the DBT mode and improved small detail detectability, especially for details located at greater heights above the table, quantified using a contrast-detail method. Furthermore, mass and calcification detectability within a 3D structured background improved compared with the previous generation of tube technology. Together with the reduced DBT acquisition time, FFS technology mitigates the limitations associated with source motion, leading to improved small detail detectability.

## Data Availability

The data that support the findings of this study are available upon reasonable request.
